# Supplementary material for: ESCRT-dependent STING degradation inhibits steady-state and cGAMP-induced signalling
Source: Nat Commun. 2023 Feb 4;14:611. doi: 10.1038/s41467-023-36132-9 (PMC9899276; doi:10.1038/s41467-023-36132-9)
Supplement: Supplementary file 1 — Supplementary Information [file 41467_2023_36132_MOESM1_ESM.pdf]

SUPPLEMENTARY MATERIALS

ESCRT-dependent STING degradation inhibits steady-state and cGAMP-induced signalling

Matteo Gentili[1], Bingxu Liu[1,2,3], Malvina Papanastasiou[1], Deborah Dele-Oni[1], Marc A Schwartz[1,4,5,6], Rebecca J. Carlson[1,7], Aziz Al'Khafaji[1], Karsten Krug[1], Adam Brown[1], John G Doench[1], Steven A Carr[1], Nir Hacohen[1,8,9]

[1]Broad Institute of MIT and Harvard, Cambridge, MA, USA
[2]Department of Biology, Massachusetts Institute of Technology, Cambridge, Massachusetts, USA
[3]The Koch Institute for Integrative Cancer Research at MIT, Cambridge, Massachusetts, USA
[4]Department of Pediatrics, Harvard Medical School, Boston, Massachusetts, USA
[5]Division of Hematology/Oncology, Boston Children's Hospital, Boston, Massachusetts, USA
[6]Department of Pediatric Oncology, Dana Farber Cancer Institute, Boston, Massachusetts, USA
[7]Massachusetts Institute of Technology, Department of Health Sciences and Technology, Cambridge, Massachusetts, USA
[8]Department of Medicine, Massachusetts General Hospital, Harvard Medical School, Boston, Massachusetts, USA
[9]Center for Cancer Research, Massachusetts General Hospital, Boston, Massachusetts 02114, USA
*Correspondence: nhacohen@mgh.harvard.edu (N.H.)

Figure S1

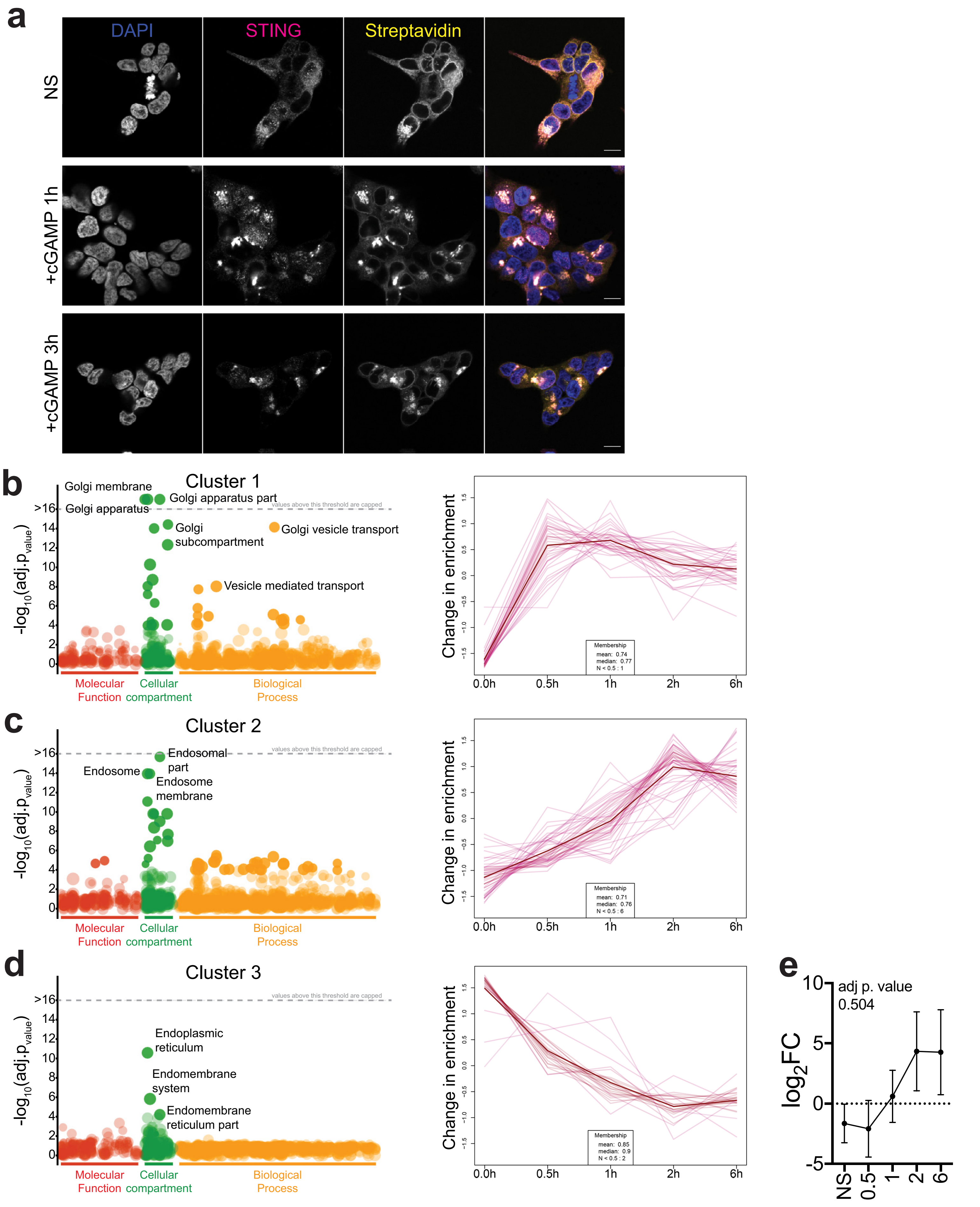
a
DAPI
STING
Streptavidin
NS
+cGAMP 1h
+cGAMP 3h
b
Cluster 1
Golgi membrane
Golgi apparatus part
Golgi apparatus
Golgi
subcompartment
Golgi vesicle transport
Vesicle mediated transport
values above this threshold are capped
-log10(adj.pvalue)
Molecular Function
Cellular compartment
Biological Process
Change in enrichment
Membership
mean: 0.74
median: 0.77
N < 0.5 : 1
0.0h
0.5h
1h
2h
6h
c
Cluster 2
Endosomal part
Endosome
Endosome membrane
Membership
mean: 0.71
median: 0.76
N < 0.5 : 6
d
Cluster 3
Endoplasmic reticulum
Endomembrane system
Endomembrane reticulum part
Membership
mean: 0.85
median: 0.9
N < 0.5 : 2
e
adj p. value
0.504
log2FC
NS
0.5
1
2
6

Figure S1. Related to Figure 1. a) Immunofluorescence of DAPI (blue) STING-TurboID (magenta) and Streptavidin-Cy5 (yellow) at the indicated time-points in 293T STING-TurboID cells post cGAMP stimulation or non-stimulated (NS). One representative field of n≥3 fields. Scale bar is 10μm. **b)** Cluster 1 from cluster analysis. Molecular Function, Cellular compartment and Biological process GO terms enrichment (left) and plot of change in enrichment of statistically significant proteins at the indicated time-points. **c)** Same as in b) for Cluster 2. **d)** Same as in b) for Cluster 3. **e)** log2FC of p62 at the indicated time-points. Line represents average and error bars SD.

Figure S2

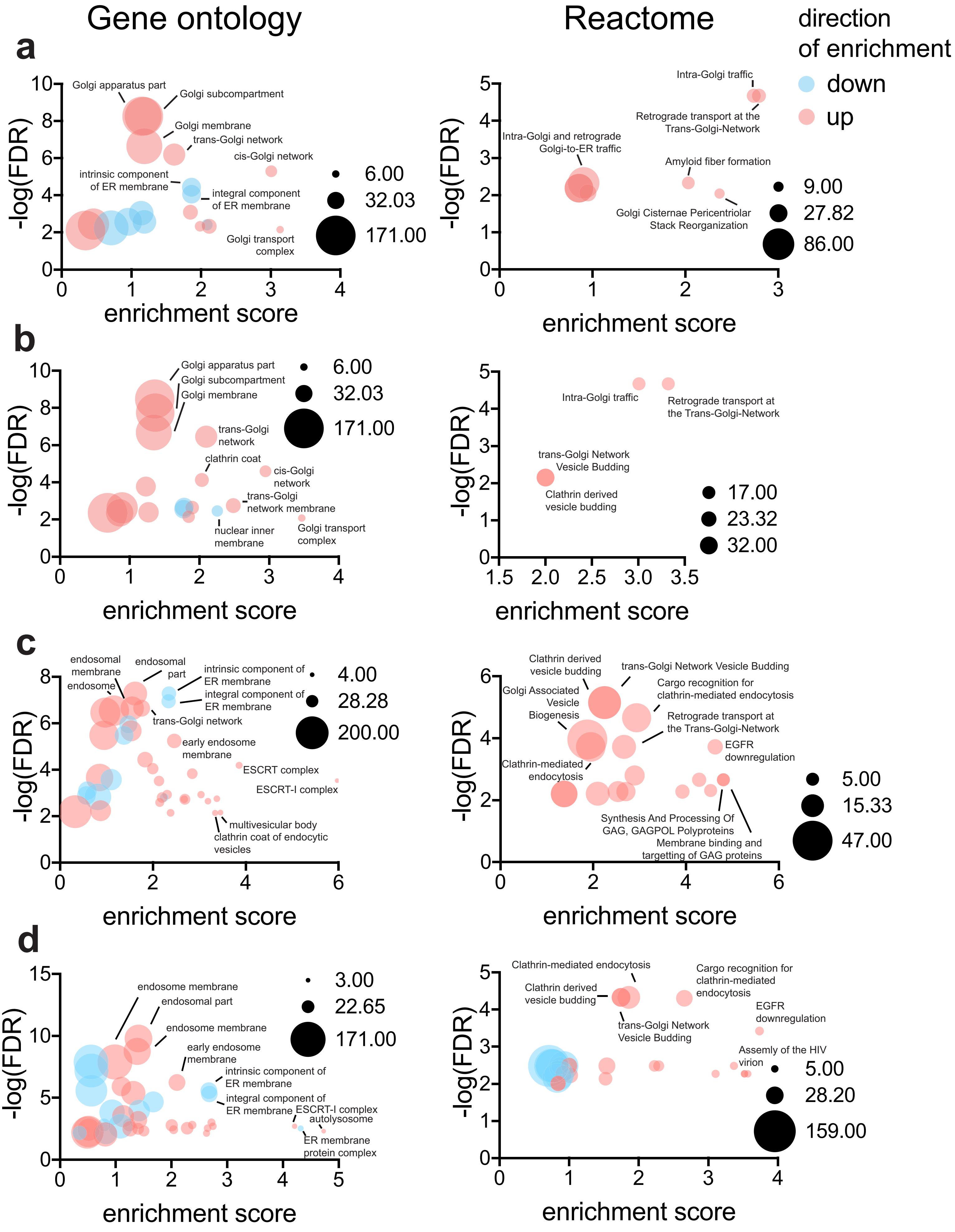
Gene ontology
Reactome
direction of enrichment
down
up
a
Golgi apparatus part
Golgi subcompartment
Golgi membrane
trans-Golgi network
cis-Golgi network
intrinsic component of ER membrane
integral component of ER membrane
Golgi transport complex
6.00
32.03
171.00
-log(FDR)
enrichment score
Intra-Golgi traffic
Retrograde transport at the Trans-Golgi-Network
Intra-Golgi and retrograde Golgi-to-ER traffic
Amyloid fiber formation
Golgi Cisternae Pericentriolar Stack Reorganization
9.00
27.82
86.00
b
clathrin coat
cis-Golgi network
trans-Golgi network membrane
nuclear inner membrane
trans-Golgi Network Vesicle Budding
Clathrin derived vesicle budding
17.00
23.32
32.00
c
endosomal membrane
endosome
endosomal part
intrinsic component of ER membrane
integral component of ER membrane
trans-Golgi network
early endosome membrane
ESCRT complex
ESCRT-I complex
multivesicular body
clathrin coat of endocytic vesicles
4.00
28.28
200.00
Golgi Associated Vesicle Biogenesis
Cargo recognition for clathrin-mediated endocytosis
EGFR downregulation
Clathrin-mediated endocytosis
Synthesis And Processing Of GAG, GAGPOL Polyproteins
Membrane binding and targetting of GAG proteins
5.00
15.33
47.00
d
endosome membrane
ESCRT-I complex
autolysosome
ER membrane protein complex
3.00
22.65
171.00
Assemly of the HIV virion
5.00
28.20
159.00

Figure S2. Related to Figure 1. Gene Ontology (GO) (left panels) and Reactome (right panels) enrichments calculated through STRING for the full TurboID dataset at **a)** 30 minutes, **b)** 1 hour, **c)** 2 hours and **d)** 6 hours post cGAMP stimulation. Terms that are positively enriched are in red, terms that are negatively enriched are in blue. Size of bubbles represent the number of genes mapped in each category. FDR: False Discovery Rate.

Figure S3

a

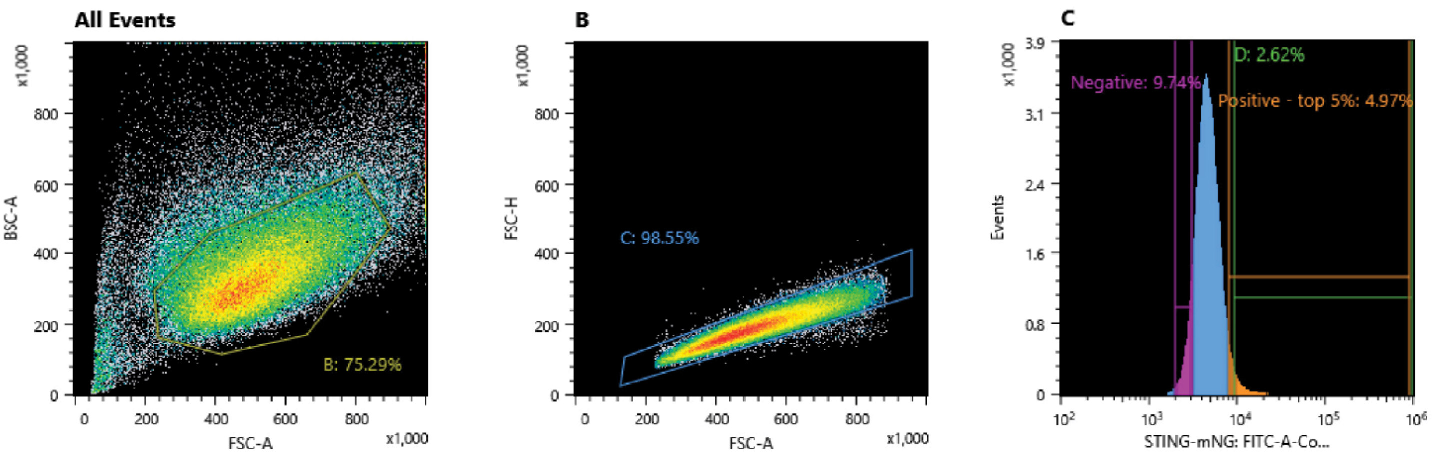

b

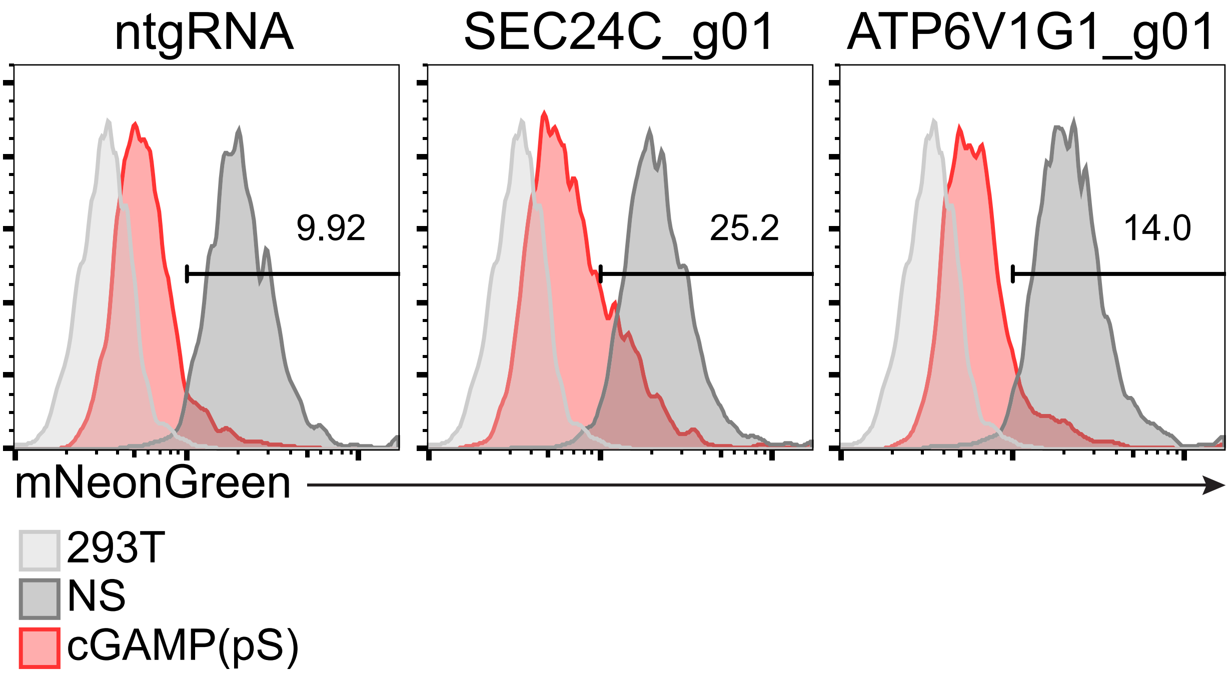

Figure S3. Related to Figure 2. a) Gating strategy for the genome-wide CRISPR screen. Gate-D in Panel C not used. **b)** mNeonGreen levels in reporter spCas9 expressing 293T STING-mNeonGreen cell lines transduced with the indicated sgRNAs before (dark gray – NS) or after (red) stimulation with 1µg/ml 2'3'-cGAMP(pS)2 for 24 hours. Line represents gating strategy, and numbers represent %STING-mNeon-Green positive cells post stimulation. 293T (light gray) are shown as a reference for mNeonGreen negative cells. One representative plot of n=2 independent experiments with n=2 technical replicates per experiment.

Figure S4

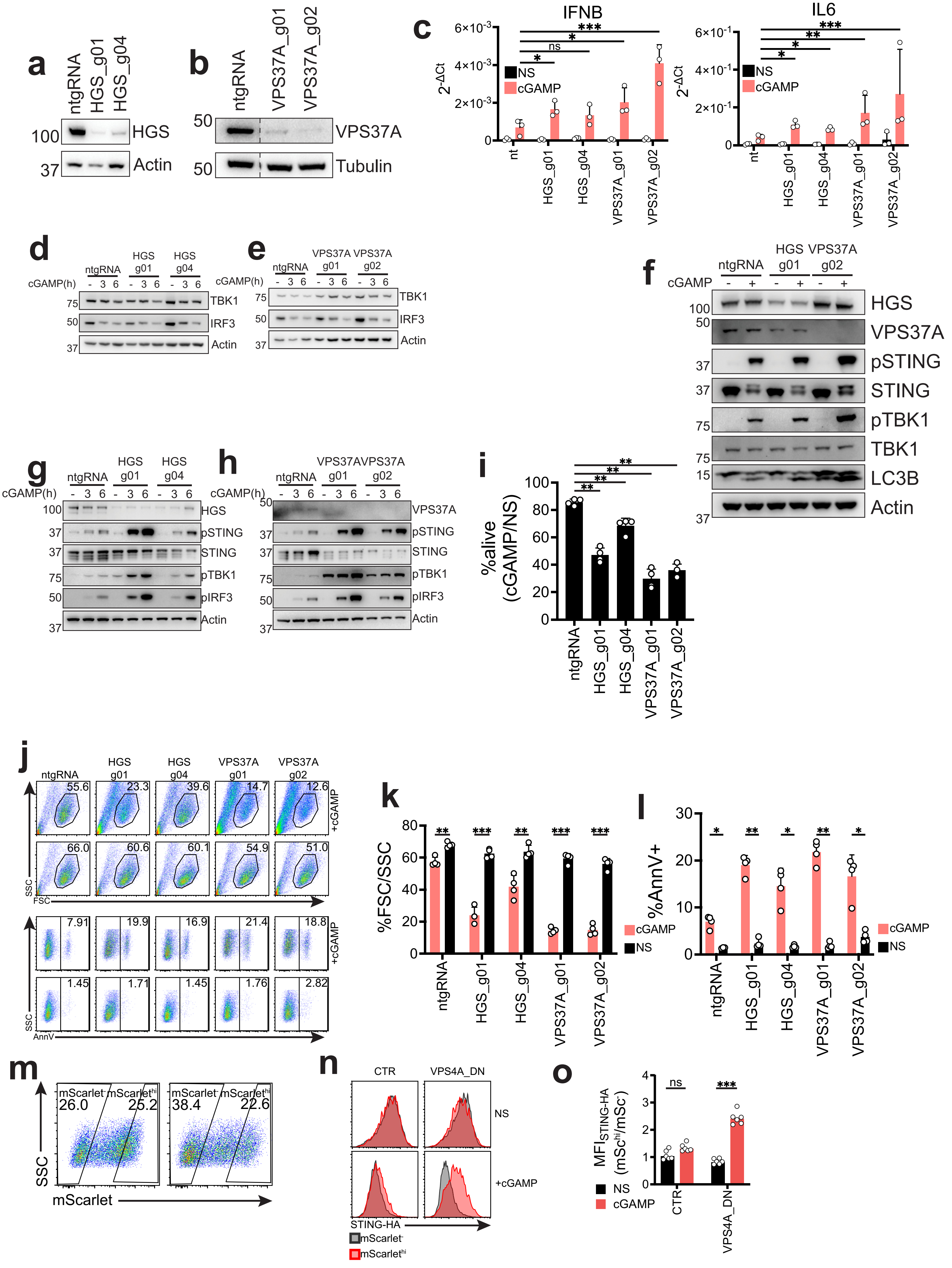
a
ntgRNA
HGS_g01
HGS_g04
HGS
Actin
b
VPS37A_g01
VPS37A_g02
VPS37A
Tubulin
c
IFNB
IL6
NS
cGAMP
2-ΔCt
ns
nt
d
e
f
cGAMP(h)
TBK1
IRF3
pSTING
STING
pTBK1
LC3B
g
h
pIRF3
i
%alive (cGAMP/NS)
j
+cGAMP
SSC
FSC
AnnV
k
%FSC/SSC
l
%AnnV+
m
mScarlet
n
CTR
VPS4A_DN
STING-HA
+cGAMP
o
MFI STING-HA (mSchi/mSc-)

Figure S4. Related to Figure 3. a) HGS KO efficiency relative to Fig. 3b, 3c. **b)** VPS37A KO efficiency relative to Fig. 3b, 3c. **c)** $2^{-\Delta Ct}$ values related to Fig. 3j. n=3 independent experiments. One-way ANOVA on log-transformed data with Dunnet multiple comparison test. **d)** Immunoblot of the indicated proteins in BJ1 KO for HGS, relative to 3h. **e)** Same as in d) for VPS37A, relative to Fig. 3i. **f)** Immunoblot of the indicated proteins in primary non-hTERT immortalized BJ fibroblasts stimulated with 0.5µg/ml cGAMP (in perm buffer) for 6h. One representative experiment of n=3 experiments. **g)** Immunoblot of the indicated proteins in U937 KO for HGS stimulated with 20µg/ml cGAMP stimulation (in medium) for 6 hours. One representative blot of n=3 independent experiments. **h)** Same as in g), for VPS37A. **i)** U937 cells were treated with 20µg/ml cGAMP (in medium) for 24 hours and alive cells quantified with Cell Titer Glo. Alive cells shown as percentage ratio RLU in cGAMP treated samples over non-stimulated samples. n=4 independent experiments. One-Way ANOVA with Dunnet multiple comparison test. **j)** FSC/SSC and Annexin V for U937 treated as in i). One representative experiment of n=4 independent experiments. **k)** Percentage of cells in FSC/SSC gate for cells treated as in j). n=4 independent experiments. One-Way ANOVA with Holm-Sidak multiple comparison test. **l)** Percentage Annexin V positive cells in FSC/SSC gate for cells treated as in j). n=4 independent experiments. One-Way ANOVA with Holm-Sidak multiple comparison test. **m)** Expression of mScarlet-VPS4ADN (VPS4A E228Q) after transfection in 293T stably expressing STING-HA and gating strategy for $mScarlet^{-}$ and $mScarlet^{hi}$ cells. **n)** STING-HA levels in cells as in n) in $mScarlet^{-}$ (dark grey) and $mScarlet^{hi}$ (red) populations that were either non-stimulated (NS) or treated with 2µg/ml cGAMP (in perm buffer) for 6 hours. One representative plot of n=3 independent experiments with n=2 technical replicates per experiment. **o)** MFI of STING-HA signals shown as a ratio of MFI of the $mScarlet^{hi}$ population over the MFI of the $mScarlet^{-}$ population. n=3 independent experiments with n=2 technical replicates per experiment. Each dot represents an individual replicate. One-way ANOVA with Dunnet multiple comparisons test.

In all panels, bar plots show mean and error bars standard deviation. Marker unit for Westen blots is KDa. *p<0.05, **p<0.01, ***p<0.001, ****p<0.0001 ns=not significant.

Figure S5

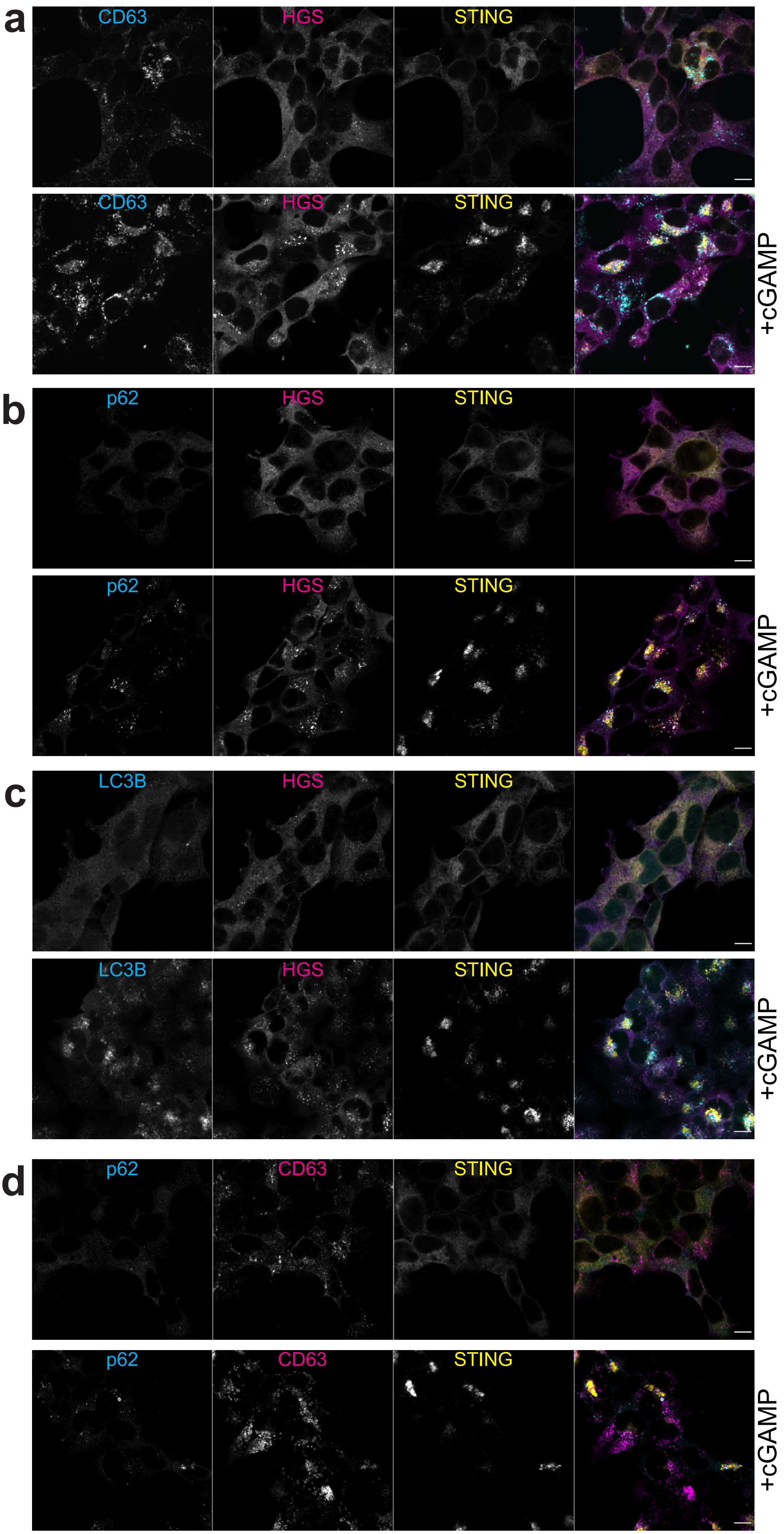
a
CD63
HGS
STING
CD63
HGS
STING
+cGAMP
b
p62
HGS
STING
p62
HGS
STING
+cGAMP
c
LC3B
HGS
STING
LC3B
HGS
STING
+cGAMP
d
p62
CD63
STING
p62
CD63
STING
+cGAMP

Figure S5. Related to Figure 4. Single color and merge of immunofluorescence of **a)** CD63 (cyan), HGS (magenta) and STING (yellow), **b)** p62 (cyan), HGS (magenta) and STING (yellow), **c)** LC3B (cyan), HGS (magenta) and STING (yellow), **d)** p62 (cyan), CD63 (magenta) and STING (yellow) in 293T stably expressing STING-HA non-stimulated or stimulated with cGAMP relative to Figure 4a-d. Scale bar is 10μm. One representative field of n≥3 independent fields in n=3 independent experiments.

Figure S6

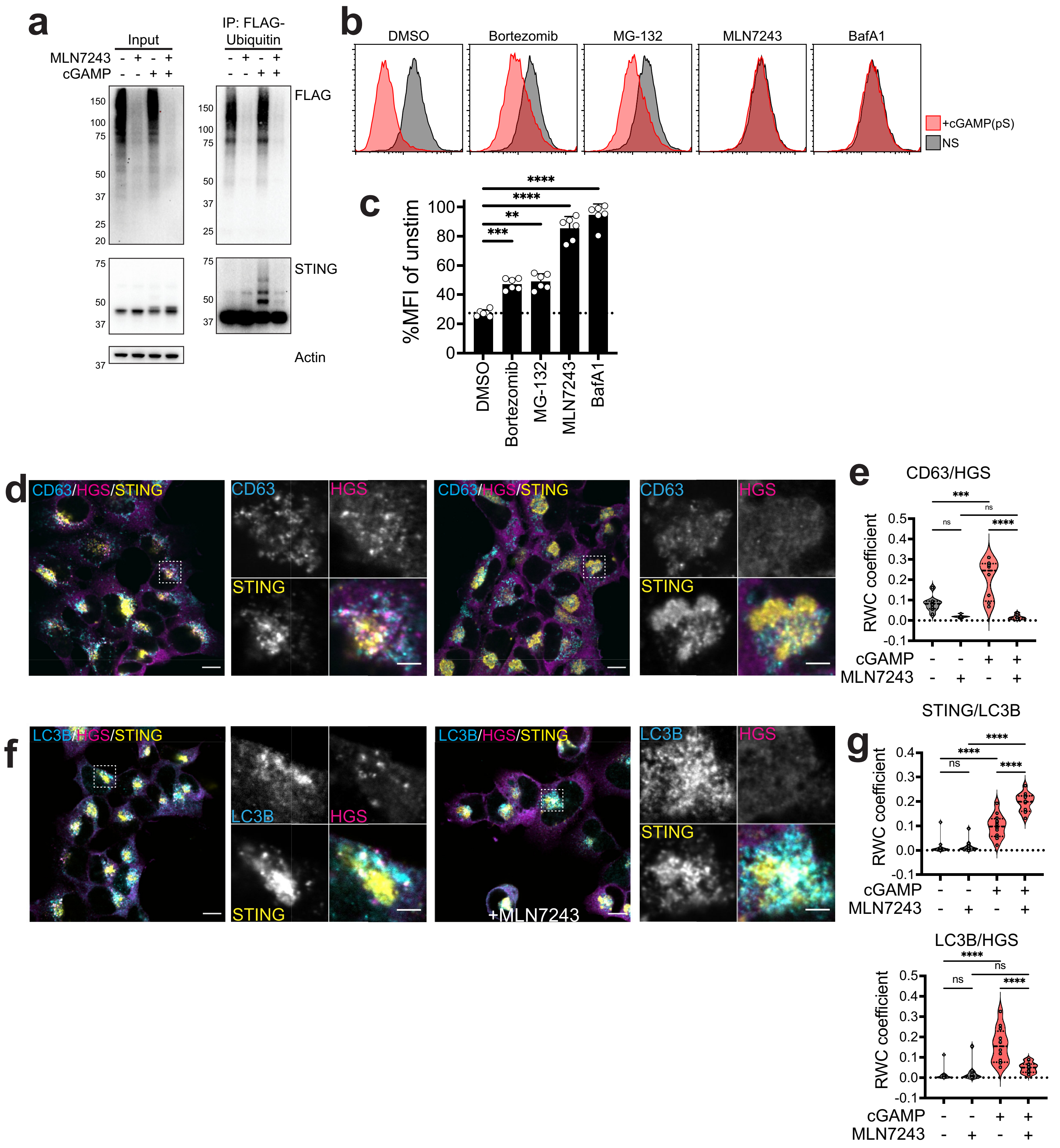
a
Input
IP: FLAG-Ubiquitin
MLN7243 - + - +
cGAMP - - + +
FLAG
STING
Actin
b
DMSO
Bortezomib
MG-132
MLN7243
BafA1
+cGAMP(pS)
NS
c
%MFI of unstim
DMSO
Bortezomib
MG-132
MLN7243
BafA1
d
CD63/HGS/STING
CD63
HGS
STING
e
CD63/HGS
RWC coefficient
cGAMP
MLN7243
f
LC3B/HGS/STING
LC3B
+MLN7243
g
STING/LC3B
LC3B/HGS

Figure S6. Related to Figure 5. a) Immunoblot of the indicated proteins in 293T stably expressing STING-HA and FLAG-ubiquitin in the input or after FLAG pulldown (IP: Flag-Ubiquitin) for cells stimulated with 2μg/ml cGAMP (in perm buffer) with 0.5μM MLN7243 for 2 hours. One representative blot of n=2 independent experiments. **b)** STING-mNG levels in 293T STING-mNG cells treated with 4μg/ml cGAMP(pS)2 (in medium) for 6 hours in presence of the indicated drugs. One representative experiment of n=3 independent experiments with n=2 technical replicates. **c)** Percentage MFI of STING-mNG signal expressed as ratio MFI of stimulated cells as in b) over MFI of unstimulated cells. n=3 independent experiments with n=2 technical replicates. Error bars represent SD. One-way ANOVA with Dunnet multiple comparisons test. **d)** Immunofluorescence of CD63 (cyan), HGS (magenta) and STING (yellow) in 293T stably STING-HA stimulated with 2μg/ml cGAMP (in perm buffer) and 0.5μM MLN7243 for 2 hours. Dashed boxes represent the cropped regions shown in the right panels. One representative field of n≥5 fields in n=2 independent experiments. Scale bar is 10μm for wide-field and 3μm for enlargement. Control non-stimulated cells are in Fig. S7. **e)** RWC for CD63 and HGS in cells stimulated as in d). Each dot represents colocalization calculated in a field. n=2 independent experiments with n≥5 fields. One way ANOVA with post-hoc Tukey test. **f)** Immunofluorescence of CD63 (cyan), HGS (magenta) and STING in cells treated as in d). Dashed boxes represent the cropped regions shown in the right panels. One representative field of n≥5 fields in n=2 independent experiments. Scale bar is 10μm for wide-field and 3μm for enlargement. Control non-stimulated cells are in Fig. S7. **g)** RWC for LC3B and STING colocalization or LC3B and HGS colocalization in cells stimulated as in f). Each dot represents colocalization calculated in a field. n=2 independent experiments with n≥5 fields. One way ANOVA with post-hoc Tukey test.

In all panels, bar plots show mean and error bars standard deviation. Marker unit for Westen blots is KDa. *p<0.05, **p<0.01, ***p<0.001, ****p<0.0001 ns=not significant.

Figure S7

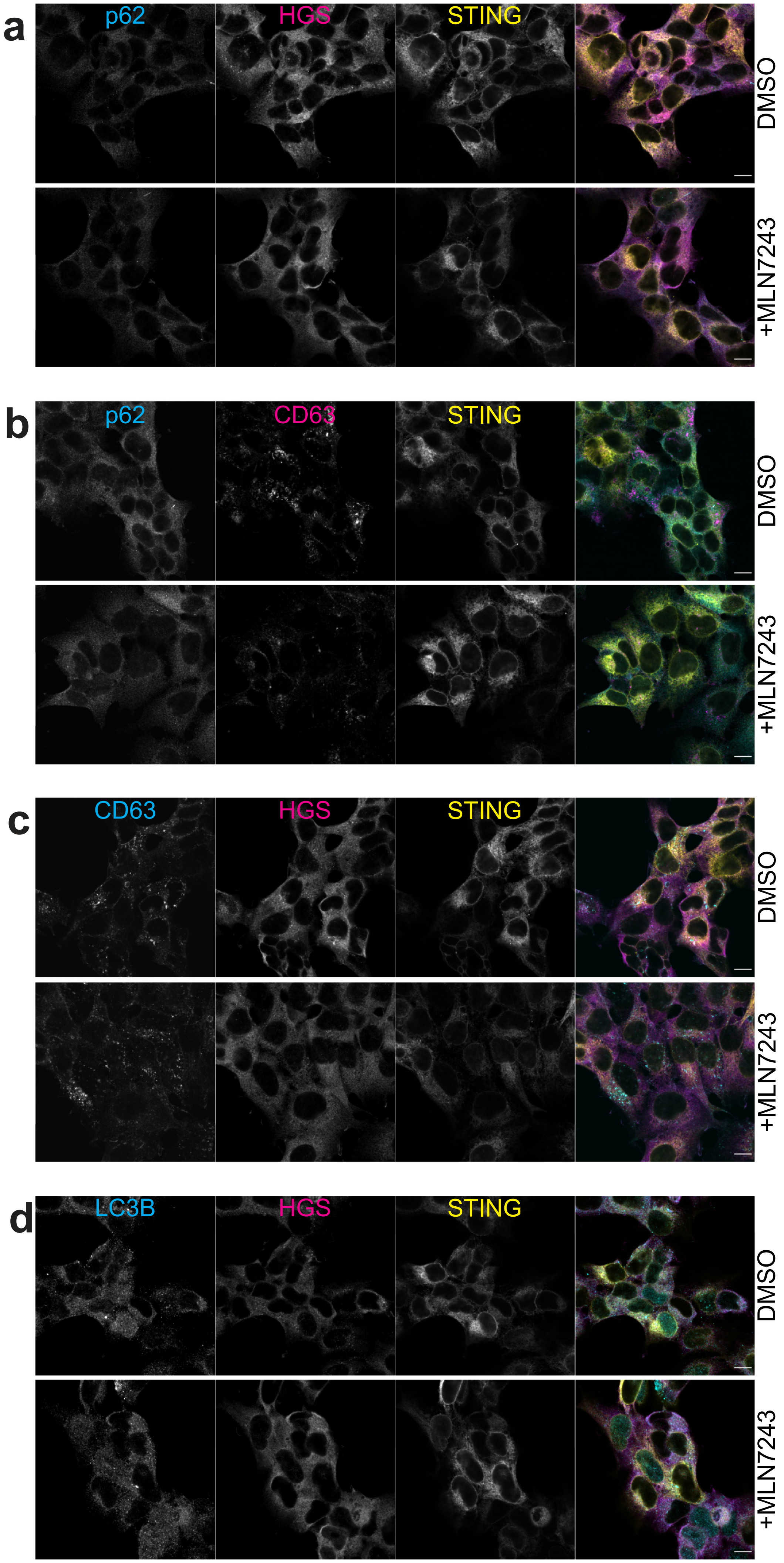
a
p62
HGS
STING
DMSO
+MLN7243
b
p62
CD63
STING
DMSO
+MLN7243
c
CD63
HGS
STING
DMSO
+MLN7243
d
LC3B
HGS
STING
DMSO
+MLN7243

Figure S7. Related to Figure 5 and Figure S6. Immunofluorescence of **a)** p62 (cyan), HGS (magenta) and STING (yellow), **b)** p62 (cyan), CD63 (magenta) and STING (yellow), **c)** CD63 (cyan), HGS (magenta) and STING (yellow), **d)** LC3B (cyan), CD63 (magenta) and STING (yellow) in 293T stably expressing STING-HA treated (bottom panels) or non-treated (DMSO – top panels) with MLN7243.

Figure S8

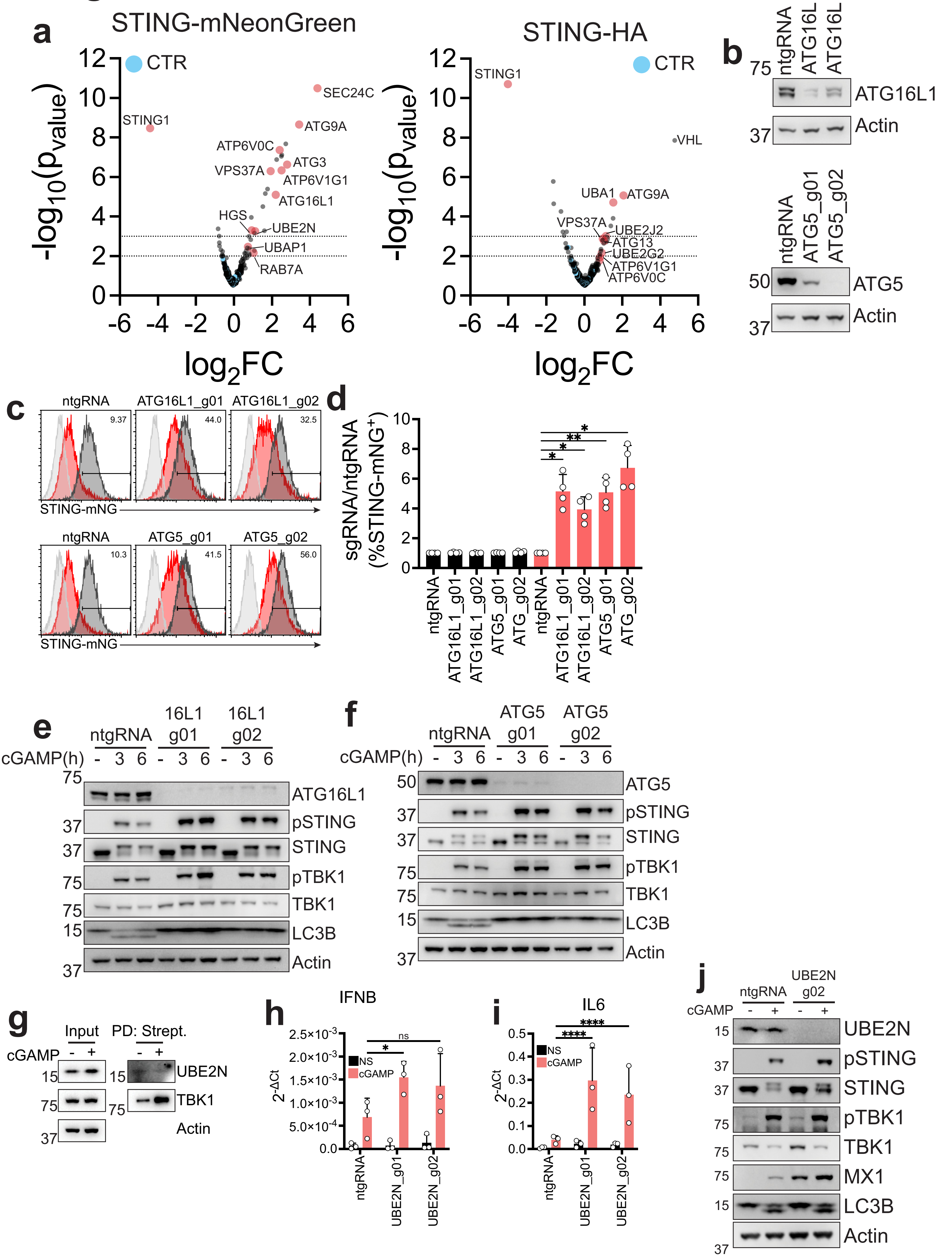
a
STING-mNeonGreen
STING-HA
CTR
-log10(pvalue)
log2FC
STING1
SEC24C
ATG9A
ATP6V0C
ATG3
VPS37A
ATP6V1G1
ATG16L1
HGS
UBE2N
UBAP1
RAB7A
VHL
UBA1
UBE2J2
ATG13
UBE2G2
ATP6V1G1
ATP6V0C
b
ntgRNA
ATG16L1_g01
ATG16L1_g02
ATG16L1
Actin
ATG5_g01
ATG5_g02
ATG5
c
STING-mNG
d
sgRNA/ntgRNA (%STING-mNG+)
e
cGAMP(h)
16L1 g01
16L1 g02
pSTING
STING
pTBK1
TBK1
LC3B
f
ATG5 g01
ATG5 g02
g
Input
PD: Strept.
cGAMP
UBE2N
TBK1
h
IFNB
NS
ns
UBE2N_g01
UBE2N_g02
i
IL6
j
UBE2N g02
MX1

Figure S8. Related to Figure 6. a) Volcano-plos of log_2 fold change (log_2FC) vs $-log_{10}(p_{value})$ after sequencing and analysis of the targeted screens in STING-mNeonGreen and STING-HA cell lines. VHL scoring highly in the STING-HA screen is due to the use of a hPGK promoter to drive STING-HA expression. KO of VHL stabilizes HIF1α which drives transcription from the HRE elements present in the hPGK promoter. **b)** Immunoblot of the indicated proteins in 293T STING-mNG KO for the indicated genes. **c)** mNG levels in 293T STING-mNG cells lines same as in b) stimulated with 4μg/ml 2'3'-cGAMP(pS)2 (in medium) for 6 hours. One representative plot of n=2 independent experiments with n=2 technical replicates per experiment. **d)** Percentage of STING-mNG positive cells in cells stimulated as in c). n=2 independent experiments with n=2 technical replicates per experiment. Each dot represents an individual replicate. Error bars represent SD. One-way ANOVA with Dunnet multiple comparison test. **e)** Immunoblot of the indicated proteins in BJ1 fibroblasts KO for ATG16L1. One representative experiment of n=2 independent experiments. **f)** Immunoblot of the indicated proteins in BJ1 fibroblasts KO for ATG5. One representative experiment of n=2 independent experiments. Marker unit is KDa. **g)** Immunoblot of the indicated proteins in 293T STING-TurboID stimulated for 1 hour with 2μg/ml cGAMP (in perm buffer). **h)** $2^{-\Delta Ct}$ values related to Fig. 6f. **i)** and Fig. 6g. n=3 independent experiments. One-way ANOVA on log-transformed data with Dunnet multiple comparison test. **j)** Immunoblot of the indicated proteins in primary non-hTERT immortalized BJ fibroblasts stimulated with 0.5μg/ml cGAMP (in perm buffer) for 6h. One representative experiment of n=2 experiments.

In all panels, bar plots show mean and error bars standard deviation. Marker unit for Westen blots is KDa. *p<0.05, **p<0.01, ***p<0.001, ****p<0.0001 ns=not significant.

Figure S9

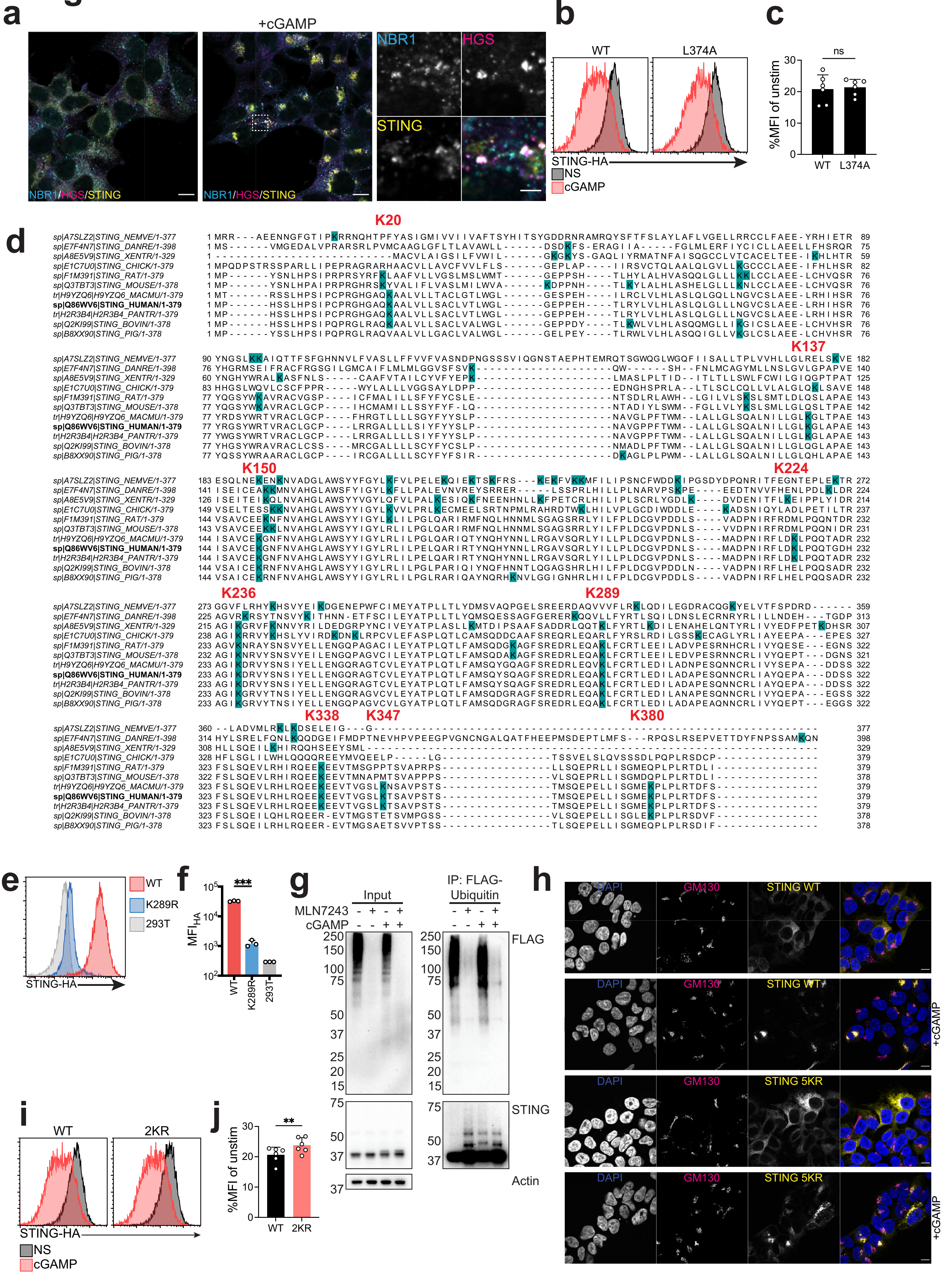

a
+cGAMP
NBR1
HGS
STING
NBR1/HGS/STING
b
WT
L374A
STING-HA
NS
cGAMP
c
%MFI of unstim
ns
WT
L374A
d
K20
K137
K150
K224
K236
K289
K338
K347
K380
sp|A7SLZ2|STING_NEMVE/1-377
sp|E7F4N7|STING_DANRE/1-398
sp|A8E5V9|STING_XENTR/1-329
sp|E1C7U0|STING_CHICK/1-379
sp|F1M391|STING_RAT/1-379
sp|Q3TBT3|STING_MOUSE/1-378
tr|H9YZQ6|H9YZQ6_MACMU/1-379
sp|Q86WV6|STING_HUMAN/1-379
tr|H2R3B4|H2R3B4_PANTR/1-379
sp|Q2KI99|STING_BOVIN/1-378
sp|B8XX90|STING_PIG/1-378
e
WT
K289R
293T
STING-HA
f
MFI_{HA}
***
WT
K289R
293T
g
Input
IP: FLAG-Ubiquitin
MLN7243
cGAMP
FLAG
STING
Actin
h
DAPI
GM130
STING WT
STING 5KR
+cGAMP
i
WT
2KR
STING-HA
NS
cGAMP
j
%MFI of unstim
**
WT
2KR

Figure S9. Related to Figure 7. a) Immunofluorescence of NBR1 (cyan), HGS (magenta) and STING (yellow) in 293T STING-HA cells stimulated with 2µg/ml cGAMP (in perm buffer) for 2h. Dashed boxes represent the cropped regions shown in the right panels. One representative field of n≥5 fields in n=2 independent experiments. Scale bar is 10µm for wide-field and 3µm for enlargement. **b)** HA level in 293T cells expressing STING WT-HA or STING L374A-HA stimulated with 2µg/ml cGAMP (in perm buffer) for 6h. One experiment representative of n=3 independent experiments with n=2 technical replicates. **c)** MFI of cells as in b) shown as %MFI of cGAMP stimulated over non-stimulated (NS) for each mutant. n=3 independent experiments with n=2 technical replicates per experiment. Each dot represents an individual replicate. Two-tailed paired t-test. **d)** Alignment of STING in different species. Lysines are highlighted and red numbers refer to positions in human STING. **e)** HA levels in 293T stably expressing HA tagged STING WT or K289R. One representative plot of n=3 technical replicates. **f)** MFI for cells as in e). Each dot represents one technical replicate. Two tailed paired t-test. **g)** Immunoblot of the indicated proteins in 293T expressing STING 5KR-HA and FLAG-ubiquitin in the input or after FLAG pulldown (IP: Flag-Ubiquitin) stimulated with 2µg/ml cGAMP (in perm buffer) with 0.5µM MLN7243 for 2 hours. One representative blot of n=2 independent experiments. Marker unit is KDa. **h)** Immunofluorescence of DAPI (blue), GM130 (magenta) and STING (yellow) in 293T stably expressing either STING WT or STING 5KR stimulated with 2µg/ml cGAMP (in perm buffer) for 2h. One representative field of n=3 fields for n=2 independent experiments. Scale bar is 20µm. **i)** HA levels in 293T expressing STING WT or STING 2KR (K338R/K370R) stimulated with 2µg/ml cGAMP (in perm buffer) for 6h. One representative experiment of n=3 independent experiments with n=2 technical replicates. **k)** MFI of cells as in i) shown as %MFI of cGAMP stimulated over non-stimulated (NS) for each mutant. n=3 independent experiments with n=2 technical replicates per experiment. Each dot represents an individual replicate. Two-tailed paired t-test.

In all panels, bar plots show mean and error bars standard deviation. Marker unit for Westen blots is KDa. *$p<0.05$, **$p<0.01$, ***$p<0.001$, ****$p<0.0001$ ns=not significant.

Figure S10

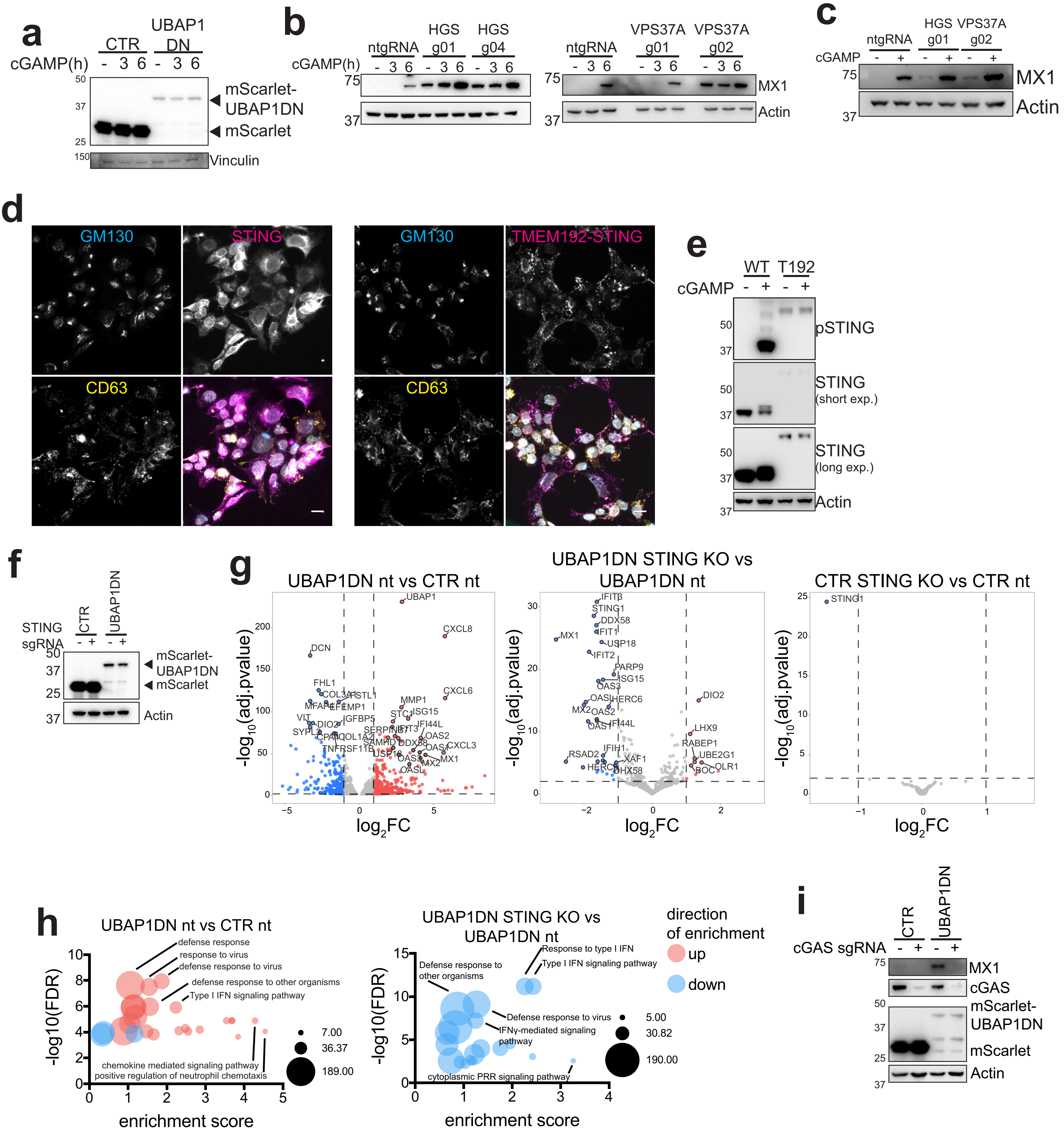
a
CTR
UBAP1 DN
cGAMP(h)
mScarlet-UBAP1DN
mScarlet
Vinculin
b
ntgRNA
HGS g01
HGS g04
VPS37A g01
VPS37A g02
MX1
Actin
c
cGAMP
d
GM130
STING
CD63
TMEM192-STING
e
WT
T192
pSTING
STING (short exp.)
STING (long exp.)
f
STING sgRNA
g
UBAP1DN nt vs CTR nt
UBAP1DN STING KO vs UBAP1DN nt
CTR STING KO vs CTR nt
-log10(adj.pvalue)
log2FC
h
UBAP1DN nt vs CTR nt
defense response
response to virus
defense response to virus
defense response to other organisms
Type I IFN signaling pathway
chemokine mediated signaling pathway
positive regulation of neutrophil chemotaxis
-log10(FDR)
enrichment score
UBAP1DN STING KO vs UBAP1DN nt
Response to type I IFN
Type I IFN signaling pathway
Defense response to other organisms
Defense response to virus
IFNγ-mediated signaling pathway
cytoplasmic PRR signaling pathway
direction of enrichment
up
down
i
cGAS sgRNA
cGAS

Figure S10. Related to Figure 8. a) Immunoblot of the indicated proteins. Related to Fig. 8f. Marker unit is KDa. **b)** Immunoblot of the indicated proteins in BJ1 fibroblasts stimulated with 0.5µg/ml cGAMP (in perm buffer) for the indicated times. One representative experiment of n=3 experiments. **c)** Immunoblot of the indicated proteins in primary non-hTERT immortalized BJ fibroblasts stimulated with 0.5µg/ml cGAMP (in perm buffer) for 6h. One representative experiment of n=2 experiments. **d)** Immunofluorescence of GM130 (cyan), HA (magenta) and CD63 (yellow) in 293T stably expressing STING-HA (left panel) or TMEM192-STING-HA (right panel). DAPI is grey in the merged image. Scale bar is 20µm. One field representative of n≥5 fields per construct of n=2 technical replicates. **e)** Immunoblot of the indicated proteins in 293T stably expressing WT STING-HA (WT) or TMEM192-STING-HA (T192) stimulated with 2µg/ml cGAMP (in perm buffer) for 2h. One experiment representative of n=2 independent experiments with n=2 technical replicates. **f)** Immunoblot of the indicated proteins. Related to Fig. 8k. Marker unit is KDa. **g)** Volcano-plots of differentially expressed genes (DEGs) for the indicated comparisons. Dashed lines to indicate significant DEGs are drawn at $\log_2FC \geq 1$ and $\log_2FC \leq -1$ and $-\log_{10}$(adjusted p value)≥2. Significant downregulated genes are in blue, upregulated in red. nt: non-targeting sgRNA **h)** GO analysis of significantly enriched processes in the indicated conditions. Positively enriched terms are in red, negatively enriched in blue. Size of bubbles represents the number of mapped genes in each category. FDR: False Discovery Rate. **i)** Immunoblot of the indicated proteins in BJ1 expressing mScarlet (CTR) or mScarlet-UBAP1DN transduced with spCas9 and a control sgRNA or a cGAS targeting sgRNA. One experiment representative of n=3 independent experiments.

In all panels, bar plots show mean and error bars standard deviation. Marker unit for Westen blots is KDa. *p<0.05, **p<0.01, ***p<0.001, ****p<0.0001 ns=not significant.

Figure S11

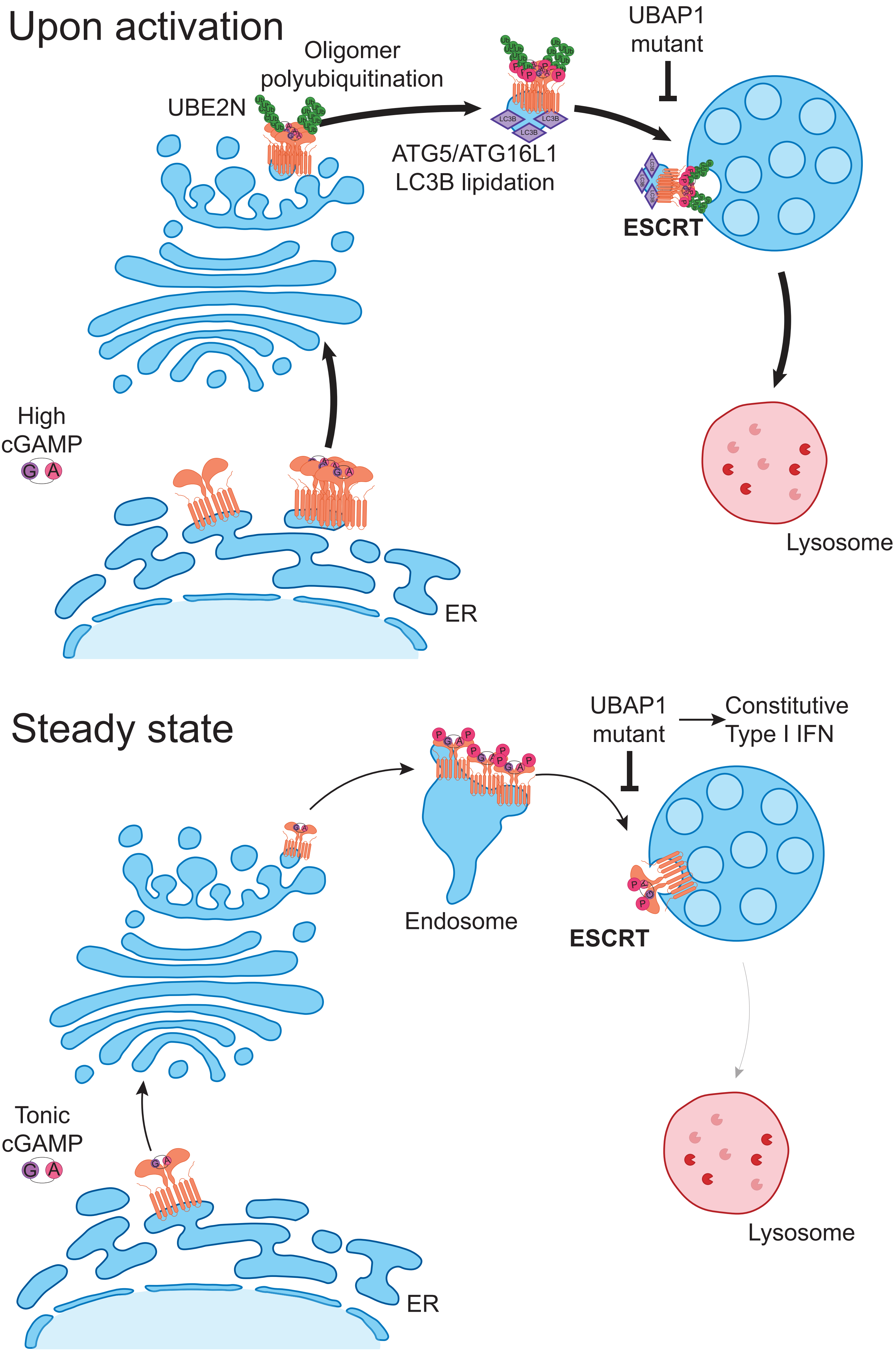
Upon activation
Oligomer polyubiquitination
UBE2N
UBAP1 mutant
ATG5/ATG16L1 LC3B lipidation
ESCRT
High cGAMP
Lysosome
ER
Steady state
UBAP1 mutant
Constitutive Type I IFN
Endosome
ESCRT
Tonic cGAMP
Lysosome
ER

Figure S11. ESCRT-dependent STING degradation curtails steady-state and cGAMP-induced signaling *Top.* Activated STING traffics from the ER to the Golgi and then to the endosome. High levels of intracellular cGAMP drive STING oligomerization which in turns lead to UBE2N dependent STING polyubiquitination and ATG5/ATG16L1 dependent LC3B lipidation of STING containing vesicles. Ubiquitination of STING drives its association with ESCRT at the late endosome. Association of ESCRT to STING creates an organizing center for fusion with the endolysosomal compartment leading to STING degradation. Pathogenic mutants of the ESCRT-I subunit UBAP1 block this process and lead to exacerbated STING responses.

Bottom. At steady state, cGAS primes tonic STING trafficking between the ER and the lysosomes through the endosomal compartment. ESCRT ensures removal of STING at steady state preventing spontaneous activation of the sensor. A UBAP1 mutant, or KO of HGS and VPS37A, blocking ESCRT function leads to accumulation of phosphorylated STING at the endosome consequently driving constitutive STING activation. Mutations in genes regulating post-Golgi STING trafficking could therefore lead to spontaneous activation of the sensor and underlie disease.

Figure S12

Gating strategy for experiments with STING-mNG cells

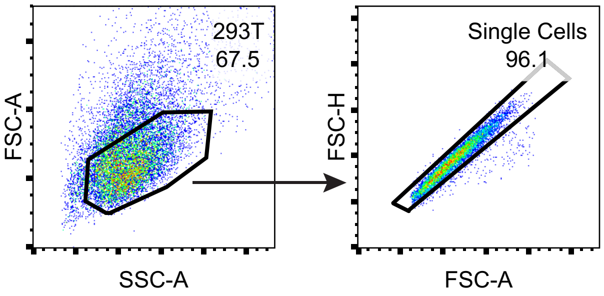

Related to Fig. 3b, 5a, 6c, 7a, S6b, S6c

Gating strategy for experiments with STING-HA cells

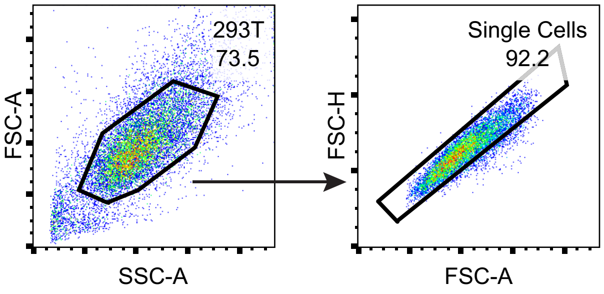

Related to Fig. 7a, 7d, 8c, S4m, S9b, S9e, S9i

Gating strategy for experiments with CD14+ monocytes

Non-stimulated cells

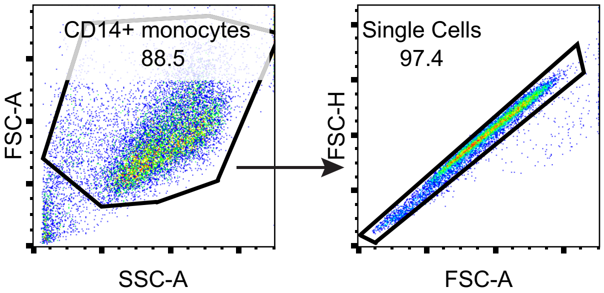

Related to Fig. 5k

cGAMP+MLN7243 stimulated cells

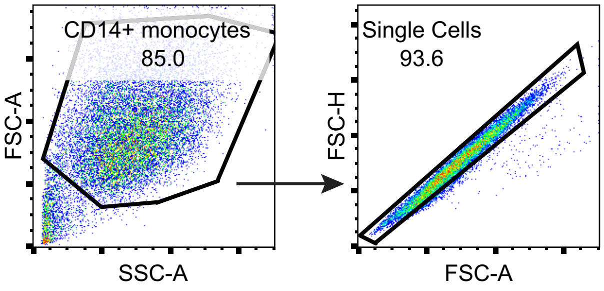

Gating strategy for experiments with MDDCs

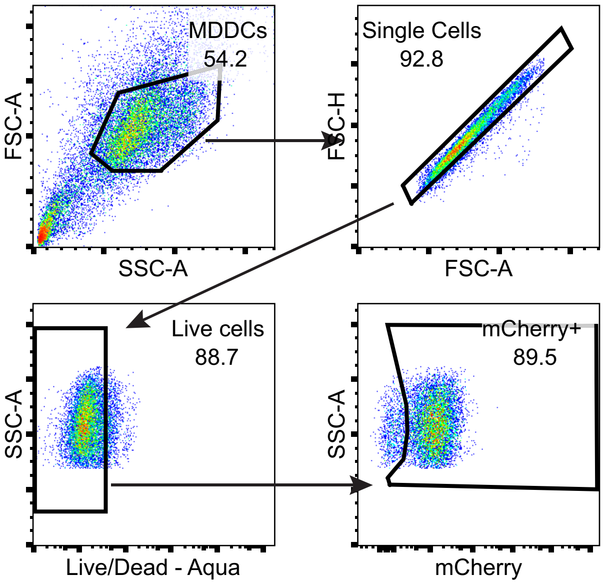

Related to Fig. 8h

Table S1. List of sgRNAs

sgRNA name	sgRNA sequence	Vector
**ntgRNA**	GTATTACTGATATTGGTGGG	CROPseq-guide-Puro or pXPR_BRD023
**SEC24C_g01**	GAAGATGACAGGAACAACCG	CROPseq-guide-Puro
**SEC24C_g02**	AAGAGCCCAGGGATAGCTCA	CROPseq-guide-Puro
**ATP6V1G1_g01**	TGAACAGTACCGCCTGCAGA	CROPseq-guide-Puro
**ATP6V1G1_g02**	TGCCACGGGATCCCAATGCC	CROPseq-guide-Puro
**HGS_g01**	CTTGGGGTACGAAGTGTACG	pXPR_BRD023
**HGS_g04**	CAGCCCCTCATAGTACACTG	pXPR_BRD023
**VPS37A_g01**	CATAAGGAGACATCCCACTT	pXPR_BRD023
**VPS37A_g02**	GGTGGATAAACACTGATCAC	pXPR_BRD023
**STING_g01**	CATTACAACAACCTGCTACG	pXPR_BRD023
**cGAS_g01**	ATCCCTCCGTACGAGAATGG	pXPR_BRD023
**UBE2N_g01**	TGGTTCTGCCAGCAAACGCT	pXPR_BRD023
**UBE2N_g02**	CTGTTGCCTTCATAGATAAG	pXPR_BRD023
**ATG16L1_g01**	GCTGCAGAGACAGGCGTTCG	pXPR_BRD023
**ATG16L1_g02**	AAAAGCATGACGTACCAAAC	pXPR_BRD023
**ATG5_g01**	AAGAAGACATTAGTGAGATA	pXPR_BRD023
**ATG5_g02**	AAATGTACTGTGATGTTCCA	pXPR_BRD023

Table S2. List of antibodies

Target	Type	Application	Source	Reference	Dilution
**STING (D2P2F)**	Rbt monoclonal	WB/IF	CST	13647S	WB: 1:2000<br>IF: 1:200
**STING**	Ms monoclonal	WB	R&D	MAB7169	1:1000
**pSTING (Ser366) (D7C3S)**	Rbt monoclonal	WB	CST	19781S	1:1000
**pSTING (Ser366) (D8K6H)**	Rbt monoclonal	IF	CST	40818S	1:50
**TBK1 (E8I3G)**	Rbt monoclonal	WB	CST	38066S	1:1000
**TBK1 (E9H5S)**	Ms monoclonal	WB	CST	51872S	1:1000
**pTBK1 (Ser172) (D52C2)**	Rbt monoclonal	WB	CST	5483S	1:1000
**IRF3 (D6I4C)**	Rbt monoclonal	WB	CST	11904S	1:1000
**pIRF3 (Ser386) (E7J8G)**	Rbt monoclonal	WB	CST	37829S	1:1000
**STAT1**	Rbt monoclonal	WB	CST	9172S	1:2000
**cGAS (D1D3G)**	Rbt monoclonal	WB	CST	15102S	1:1000
**pSTAT1 (Tyr 701) (58D6)**	Rbt monoclonal	WB	CST	9167S	1:2000
**MX1 (D3W7I)**	Rbt monoclonal	WB	CST	37849S	1:1000
**HRS (D7T5N)**	Rbt monoclonal	WB/IF	CST	15087S	WB: 1:2000<br>IF: 1:200
**LC3B**	Rbt monoclonal	WB/IF	CST	2775S	WB: 1:1000<br>IF: 1:100
**p62 (D5L7G)**	Rbt monoclonal	IF	CST	88588S	1:200
**Ubc13**	Rbt monoclonal	WB	CST	4919	1:1000
**CD63 (EPR22458-280)**	Rbt monoclonal	IF	Abcam	ab252919	1:400
**CD63 (MX-49.129.5)**	Ms monoclonal	IF	Santa Cruz	sc-5275	1:400
**VPS37A (G-3)**	Ms monoclonal	WB	Santa Cruz	sc-376978	1:100
**NBR1 (4BR)**	Ms monoclonal	IF	Santa Cruz	sc-130380	1:200
**UBAP1**	Rbt polyclonal	WB	Proteintech	12385-1-AP	1:1000
**RFP (6G6)**	Ms monoclonal	WB	Chromotek	6g6-100	1:1000
**ATG5 (D5F5U)**	Rbt monoclonal	WB	CST	12994S	1:1000
**ATG16L1 (D6D5)**	Rbt monoclonal	WB	CST	8089S	1:1000
**GM130 (35/GM130)**	Ms monoclonal	IF	BD	610823	1:200

			Biosciences		
**EEA1 (14/EEA1)**	Ms monoclonal	IF	BD Biosciences	610457	1:200
**β-actin (AC-15)**	Ms monoclonal	WB	Abcam	ab6276	1:2000
**α-Tubulin (DM1A)**	Ms monoclonal	WB	Thermo Fisher	14-4502-82	1:1000
**Vinculin**	Rbt monoclonal	WB	CST	4650S	1:1000
**HRP Anti-beta Actin antibody [AC-15]**	Ms monoclonal	WB	Abcam	ab49900	1:20000
**HRP-anti β-actin (mAbcam 8226)**	Ms Monoclonal - HRP conjugate	WB	Abcam	ab20272	1:5000
**HRP-Streptavidin**	NA	WB	BioLegend	405210	1:1000
**HA**	Rat monoclonal	IF	Millipore Sigma	11867423001	1:200
**DYKDDDDK Tag (D6W5B) - FLAG**	Rabbit monoclonal	WB	CST	14793S	1:1000
**Cy5-Streptavidin**	NA	IF	Biolegend	405209	1:200
**Alexa Fluor 647 anti-HA.11**	Biolegend	FC	Biolegend	682404	1:200
**Alexa 488 anti-mouse IgG (H+L)**	Gt polyclonal	IF	Thermo Fisher	A-11029	1:200
**Alexa 555 F(ab')2 anti-Rabbit IgG**	Gt polyclonal	IF	Thermo Fisher	A-21430	1:200
**Alexa 647 anti-rat IgG (H+L)**	Gt polyclonal	IF	Thermo Fisher	A-21247	1:200
**Peroxidase AffiniPure Goat Anti-Rabbit IgG (H+L)**	Gt polyclonal	WB	Jackson Immunoresearch	111-035-144	1:5000
**Peroxidase AffiniPure Goat Anti-Mouse IgG (H+L)**	Gt polyclonal	WB	Jackson Immunoresearch	111-035-146	1:5000

Rbt: rabbit; Ms: mouse; Gt: goat.
WB: western blot; IF: immunofluorescence; FC: flow cytometry.

Table S3. List of qPCR primers

Primer name	Primer sequence
IFNB1 FWD	CAGCATCTGCTGGTTGAAGA
IFNB1 RV	CATTACCTGAAGGCCAAGGA
IL6 FWD	CCCCTGACCCAACCACAAAT
IL6 RV	ATTTGCCGAAGAGCCCTCAG
GAPDH FWD	GTCTCCTCTGACTTCAACAGCG
GAPDH RV	ACCACCCTGTTGCTGTAGCCAA

Table S4. RNA sequencing reagents and conditions

RT mix 1

Reagent	Reaction conc.	µL required
PEG 8000 (50% solution)	5%	3.2
Triton X-100 (10% solution)	0.10%	0.24
RNAse Inhibitor (40µ/µL)	0.5µ/µL	0.32
Smartseq3_OligodT30VN(100µM)	0.5µM	0.16
dNTPs (10mM/each)	0.5mM/each	1.6
mRNA template		µL for 15ng
Nuclease Free Water		Up to 24µL
Total µl		24

RT mix 2

Reagent	Reaction conc.	µL required
Tris-HCl pH 8.5 (1M)	25mM	0.8
NaCl (1M)	30mM	0.96
MgCl2 (100mM)	2.5mM	0.8
GTP (100mM)	1mM	0.32
DTT (100mM)	8mM	2.56
RNase Inhibitor (40u/µl)	0.5u/µl	0.4
TSO (100µM)	2µM	0.64
Maxima H-minus RT enzyme (200U/µl)	2u/µl	0.32
Nuclease Free Water		1.2
Total µl		8

First-strand synthesis

Temperature	Time	Cycles
42 °C	90 min	1x
50 °C	2 min	10x
42 °C	2 min	
85 °C	5 min	1x

cDNA PCR mix		
Reagent	Reaction conc.	µl per. reaction
Kapa HiFi HotStart buffer (5X)	1X	10
dNTPs (10mM/each)	0.3mM/each	1.5
MgCl2 (100mM)	0.5mM	0.25
Fwd Primer (100µM) AAO270	0.5µM	0.25
Rev Primer (10µM) AAO271	0.1µM	0.5
First-strand synthesis reaction		20
Polymerase (1U/µl)	0.02U/µl	1
Nuclease Free Water		16.5
Total µl		30

cDNA amplification		
Temperature	Time	Cycles
98 °C	3 min	1x
98 °C	20 sec	25x
65°C	30 sec	
72 °C	6 min	
72 °C	5 min	1x
4 °C	Hold	

Tagementation mix		
Reagent	Reaction conc.	µl per. reaction
Tagmentation buffer (4x)<br>(Tris-HCl pH 7.5 - 40mM, MgCl2 - 20mM, DMF- 20%)	1X	2
Amplicon Tagmentation Mix (Tn5)		0.32
UltraPure water		1.68
Total		4

**Final library amplification mix**		
Reagent	Reaction conc.	µl per. reaction
Phusion HF buffer (5X)	1X	5.6
dNTPs (10mM/each)	0.2mM/each	0.6
Phusion HF (2U/µl)	0.01U/µl	0.16
Nextera Index Primers (0.5µM)	0.1µM	6
H2O		5.64
Tagmented cDNA		10
Total µl		28

**Final library amplification**		
Temperature	Time	Cycles
72 °C	3 min	1x
98 °C	3 min	1x
98 °C	10 sec	12x
55 °C	30 sec	
72 °C	30 sec	
72 °C	5 min	1x
4 °C	Hold	
